# Phylogeographic structure of *Heteroplexis* (Asteraceae), an endangered endemic genus in the limestone karst regions of southern China

**DOI:** 10.3389/fpls.2022.999964

**Published:** 2022-10-27

**Authors:** Xianliang Zhu, Hui Liang, Haolong Jiang, Ming Kang, Xiao Wei, Lili Deng, Yancai Shi

**Affiliations:** ^1^ Guangxi Institute of Botany, Guangxi Zhuang Autonomous Region and Chinese Academy of Sciences, Guilin, China; ^2^ College of Life Science, Guangxi Normal University, Guilin, China; ^3^ South China Botanical Garden, Chinese Academy of Sciences, Guangzhou, China

**Keywords:** Compositae, demographic history, gene introgression, karst plants, SNP, species differentiation

## Abstract

Though the karst regions in south and southwest China are plant diversity hotspots, our understanding of the phylogeography and evolutionary history of the plants there remains limited. The genus Heteroplexis (Asteraceae) is one of the typical representative plants isolated by karst habitat islands, and is also an endangered and endemic plant to China. In this study, species-level phylogeographic analysis of the genus Heteroplexis was conducted using restriction site-associated DNA sequencing (RADseq). The genetic structure showed a clear phylogeographic structure consistent with the current species boundaries in the H. microcephala, H. incana, H. vernonioides, H. sericophylla, and H. impressinervia. The significant global (R = 0.37, P < 0.01) and regional (R = 0.650.95, P < 0.05) isolation by distance (IBD) signals among species indicate strong geographic isolation in the karst mountains, which may result in chronically restricted gene flow and increased genetic drift and differentiation. Furthermore, the phylogeographic structure of Heteroplexis suggested a southward migration since the last glacial period. Demographic analysis revealed the karst mountains as a refuge for Heteroplexis species. Finally, both Treemix and ABBA-BABA statistic detected significant historical gene flow between species. Significant historical gene flow and long-term stability of effective population size (Ne) together explain the high genome-wide genetic diversity among species (π = 0.05370.0838). However, the recent collapse of Ne, widespread inbreeding within populations, and restricted contemporary gene flow suggest that Heteroplexis species are probably facing a high risk of genetic diversity loss. Our results help to understand the evolutionary history of karst plants and guide conservation.

## Introduction

Phylogeography explains species differentiation in relation to current geographic distribution patterns by reconstructing phylogenies and tracing the evolutionary relationships of alleles between related species or populations (Hickerson et al., 2010). With the development of high-throughput sequencing technology, phylogeography based on large-scale genomic markers is being widely used to address global hotspot issues such as species evolution, new species formation, and biological conservation (Hu et al., 2021). In particular, restriction site-associated DNA sequencing (RADseq), which is of low cost and does not require reference genomic information to obtain a large number of genome-wide markers, has been increasingly used in phylogeographic studies (Baird et al., 2008; Brandrud et al., 2020; Spriggs and Fertakos, 2021). For example, RADseq has been used to resolve complex phylogenetic relationships and species boundaries (Rubin et al., 2012; Ferrer Obiol et al., 2021; Ye et al., 2021), assess genetic diversity and structure (Cai et al., 2020), and infer interspecific gene flow (Natola et al., 2021) and demographic history (Hellwig et al., 2020; Tao et al., 2021). The obtained genetic information is valuable for studying species differentiation and evolutionary history.

Karst in Southeast Asia is known as a world biodiversity hotspot, with a remarkably high level of species richness and endemism (Clements et al., 2006). The mountainous south and southwest regions of China is home to the largest karst landscape in the world, which is an important World Natural Heritage site (Yuan, 2001; Liang et al., 2017). Due to the complex mountainous topography and the discontinuous distribution of soils, most plants are isolated in the karst habitat islands in these regions (Porembski, 2007; Palma-Silva et al., 2011; Gao et al., 2015). As a result of their long-term adaptation to such a special habitat, many karst plants are calcicole and narrowly distributed (Qi et al., 2013; Wei et al., 2017; Liao et al., 2020). Hence, karst ecosystems are a representative model to test the assumptions of island theory in a continental environment. In the limited number of phylogeographic studies involving karst plants to date, the prevalence of allopatric speciation is demonstrated, and geographic isolation is highlighted as a major driver of population diversification (Gao et al., 2015; Wang et al., 2017; Tseng et al., 2019). These studies emphasize the unique advantages of karst plants for examining island biogeographic patterns, yet their use of low-resolution markers may limit our knowledge of the geographic evolutionary patterns of karst plants (Gao et al., 2015; Wang et al., 2017; Tseng et al., 2019).

**Figure 1:**
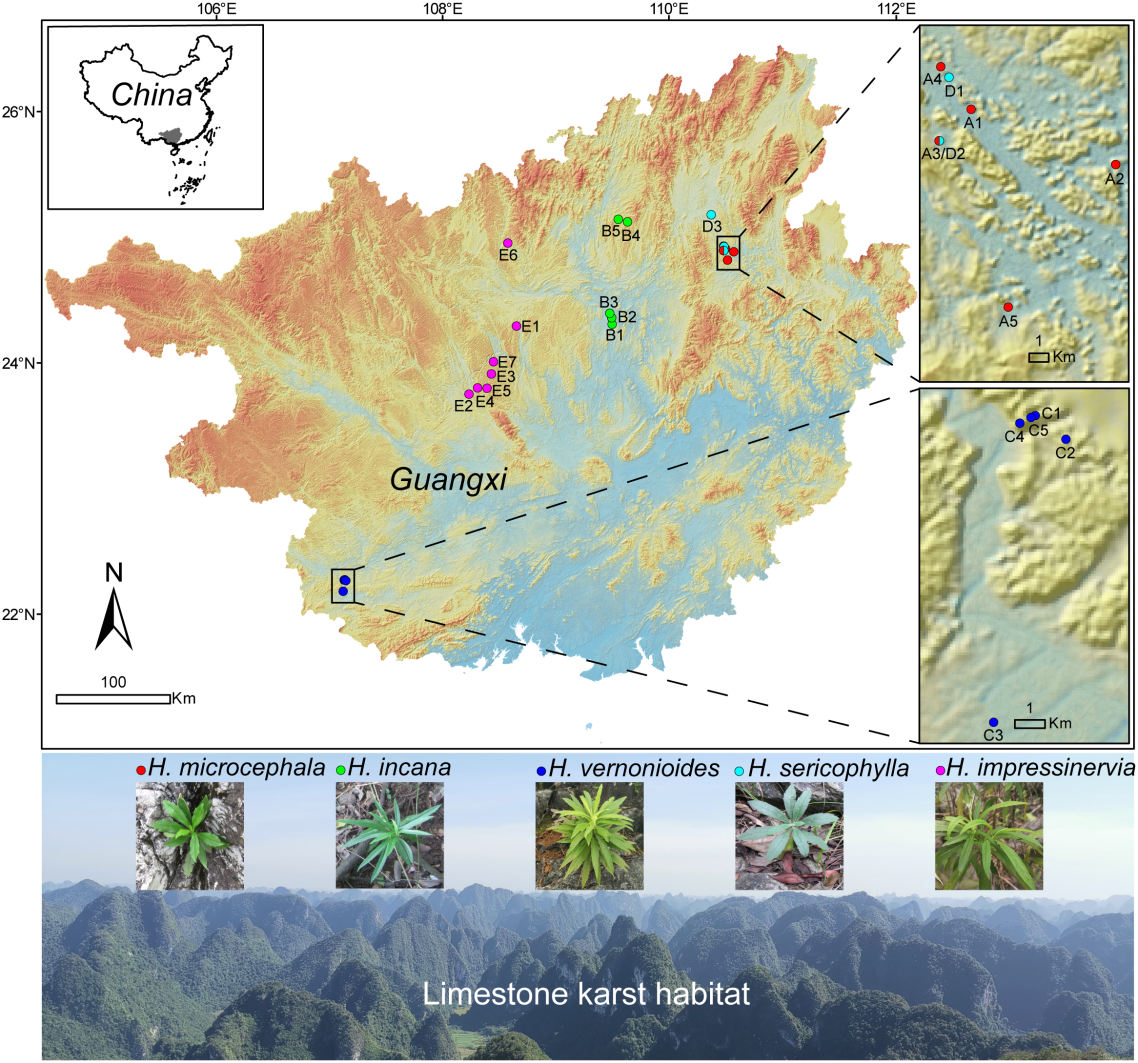
Map of the sampling sites and habitats of the five *Heteroplexis* species. Populations were color-coded by species. Site numbers correspond to the population numbers listed in **Table S1**. The map was downloaded from Geospatial Data Cloud (https://www.gscloud.cn).

The genus *Heteroplexis* C.C.Chang belongs to Trib. Astereae of the Asteraceae family (Fu et al., 2016). This genus is only narrowly distributed in karst limestone habitats, especially on mountain tops and cliffs, and is endangered and endemic to Guangxi, southern China (**Figure 1**). Among the Asteraceae family, the most special morphological feature of the genus *Heteroplexis* is the unequal corolla slivers of the hermaphrodite flower, which is also the origin of their genus name. Up to date, five Heteroplexis species have been proposed, namely *H. vernonioide* C.C.Chang, *H. microcephala* Y.L.Chen, *H. sericophylla* Y.L.Chen, and *H. incana* J.Y.Liang and *H. impressinervia* J.Y.Liang. Previous phylogenetic studies of Astereae. Previous phylogenetic studies of Astereae (Brouillet et al., 2009; Li et al., 2012) did not include the genus *Heteroplexis*, resulting in the phylogenetic relationships among *Heteroplexis* species being unclear for a long time. Until more recently, in a phylogenetic analysis of 95 species of the subfamily Asteroideae based on plastome sequence information, *H. incana* was shown to be phylogenetically close to two *Aster* species (i.e., *A. hersileoides* and *A. hypoleucus*) (Abdullah et al., 2021). However, the phylogenetic relationships within the genus *Heteroplexis* remain unresolved. In addition, except for a few populations of *H. microcephala* that have been characterized for genetic diversity using RAPD markers (Shi et al., 2016), our knowledge of the genetic differentiation and evolutionary history of the rare endemic *Heteroplexis* species to karst remains limited.

In this study, we conducted a comprehensive phylogeographic analysis of the five species of *Heteroplexis* using genome-wide SNP data. The aims were to: (1) clarify the species boundaries and phylogeographic patterns, (2) assess the effects of geographic isolation, interspecific gene flow, and demographic history on phylogeographic pattern, and (3) investigate genome-wide genetic diversity among species and provide suggestions for future conservation strategies.

## Materials and methods

### Plant materials

A total of 184 adult individuals from 25 populations of *H. microcephala* (*n* = 38), *H. incana* (*n* = 32), *H. vernonioides* (*n* = 35), *H. sericophylla* (*n* = 25), and *H. impressinervia* (*n* = 54) were sampled from the karst limestone regions of Guangxi, China (**Figure 1**; **Table S1**). Four to ten individuals per population were collected, depending on the actual number of individuals in a population. Fresh leaves were dried in silica gel and stored for later use.

### DNA extraction, RAD library preparation, and RADseq

Total genomic DNA was extracted using the CTAB method, and DNA quality was evaluated on 1% agarose electrophoresis (Doyle et al., 1996). RAD library was constructed using the restriction enzyme *EcoR1* according to the protocol of Novogene Bioinformatics Institute (Beijing, China). The final pooled libraries were transferred to a NovaSeq 6000 platform for pair-end sequencing (PE 150).

### SNP calling

The raw data were subjected to a series of quality controls using a previous script (Feng et al., 2017), including removing the adapter, reads with N ratio of more than 10%, and the low-quality reads. After that, the quality of clean reads was evaluated using Fastqc (Andrews, 2010). The *de novo* pipeline of Stacks v2.59 (Rochette et al., 2019) was then used for SNP calling. The main parameters applied were as follows: minimum depth of coverage required to create a stack (-m), 3; maximum distance (in nucleotides) allowed between stacks (-M), 5; and number of mismatches allowed between sample loci when building the catalog (-n), 3. To improve the computational efficiency, we randomly selected two individuals from each population (50 in total) when constructing the catalog using the *cstacks* module. The raw loci generated by the *gstacks* module were filtered by the *populations* module so that a high-quality polymorphic loci data set (set1) with a minimum minor allele frequency (–min-maf) of 0.02, a maximum observed heterozygosity (–max-obs-het) of 0.5, and occurrence in five species (-p) and more than 50% of the individuals per species (-r) was obtained. To reduce the bias of linkage disequilibrium (LD), a LD pruned data set (set2) which only included one random SNP per locus was produced by adding ‘–write-random-snp’ to the above parameters. Additionally, a low missing LD pruned data set (set3) of each species was generated by the parameter ‘-r 0.9 -min-maf 0.02 –max-obs-het 0.5 –write-random-snp’ for downstream analysis.

### Genetic diversity and differentiation

Genetic diversity was assessed using data set1. Nucleotide diversity (π), observed heterozygosity (*H*_O_), expected heterozygosity (*H*_E_), and inbreeding coefficient (*F*_IS_) were calculated using the *populations* module in Stacks. Genetic differentiation coefficient (*F*_ST_) and absolute sequence divergence (*D*_XY_) were calculated using the script popgenWindows.py (https://github.com/simonhmartin/genomics_general). The identity-by-state (IBS) kinship matrix between individuals was analyzed using Tassel v5.0 (Bradbury et al., 2007).

### Phylogenetic analysis

The phylogenetic relationship of the five *Heteroplexis* species was reconstructed using both the gene tree and species tree approaches. Prior to tree reconstruction, the VCF files of data set1 and set2 were converted to phylip format using the vcf2phylip.py script (Ortiz, 2019). Gene tree reconstruction was performed based on data set1 using the maximum likelihood (ML) approach in IQtree v2.0 (Minh et al., 2020). The best-fit model (GTR+F+R3) was chosen according to the Akaike information criterion (AIC); UFboot and SH-aLRT support values were calculated from 1000 replicates (Hoang et al., 2018). Additionally, a species tree was reconstructed based on data set2 under the multispecies coalescent model with SVDQuartets (Chifman and Kubatko, 2014), a novel algorithm implemented in PAUP v4.0a168 (Swofford, 2003). Branch support was assessed by randomly sampling 50,000 quartets and performing 1000 bootstrap replicates. The resulting trees were visualized and edited using Figtree v1.4.4.

### Genetic structure

Admixture v1.3.0 (Alexander et al., 2009) was used to estimate individual ancestries from *K* ancestors based on data set2. We varied *K* from 1 to 9 and selected the most likely *K* based on the minimum cross-validation (CV) error. In addition, principal component analysis (PCA), a dimensionality-reduction method, was accomplished in Plink v1.07 (Chang et al., 2015).

### Isolation by distance (IBD)

To investigate the effect of geographic distance on genetic differentiation, a Mantel test was conducted for all 25 populations using the ‘vegan’ package in R (https://github.com/vegandevs/vegan/). Correlation coefficient was calculated using the Spearman method, and 9999 permutations were performed to test for significance. A Mantel test was performed for each species as well, but *H. sericophylla* was excluded due to its small sample size (three populations).

### Interspecific gene flow

Treemix v1.13 (Pickrell and Pritchard, 2012) was used to infer the direction and strength of gene flow among *Heteroplexis* species. Data set2 was converted to format using the *populations* module in Stacks. *H. microcephala* was set as an outgroup due to its being a stable root-most clade in both the previous gene tree and the species tree, and 0 5 migration events (m) between species were assumed to construct the ML tree using Treemix. The R package OptM (Fitak, 2021) was used to determine the best m value. To further confirm the genetic introgression between species, ABBA-BABA statistic (also known as *D*-statistic) was performed using Dsuite v0.3 (Malinsky et al., 2021). The *D*-statistic is designed for a 4-taxon fixed phylogeny of Pl, P2, P3, and outgroup (O). When there is no gene introgression between O, P1, P2, and P3, the *D*-statistic would be 0. If, however, there is gene introgression between P2 and P3 or P1 and P3, the *D*-statistic would be positive or negative. The VCF file of data set2 was used directly as the input, the ML tree as an optional file, and *H. microcephala* as the outgroup. The *Dtrios* procedure was then used to identify gene introgression between species at a significant level of *P* < 0.01.

### Demographic history

Stairway Plot v2.0 (Liu and Fu, 2020) was used to fit the changes in effective population size (*N_e_*) of *Heteroplexis* species in different historical periods based on the joint site frequency spectrum (SFS). Folded SFS was calculated for each species based on the low missing LD pruned data set3 using the script easySFS.py (https://github.com/isaacovercast/easySFS). A generation time of 5 years was estimated based on our long-term field observation of the time from seeds to flowering, and as the precise SNP mutation rates in *Heteroplexis* are still unknown, a mutation rate of 8.25 × 10^-9^ per site per year was assumed according to that recently reported in asterid (Song et al., 2018). The median and confidence intervals (CIs) of *N_e_* were estimated from 200 bootstrap replicates.

## Results

### RADseq and SNP calling

After quality control, a total of 2,005,212,749 clean paired-end reads were obtained from the 184 samples with a GC content of 36.84%, Q20 of 98.68%, and Q30 of 94.67% on average (**Table S2**). Following the *de novo* pipeline of Stacks, a total of 28,641 loci with 16,566,246 sites from all samples were developed. With strict filtering parameters, data set1 retained 319,506 high-quality SNPs, and data set2 was further reduced to 27,836 SNPs through LD pruning (**Table S3**). Low missing LD pruned data set3 was also generated for each species, containing 3998 (*Heteroplexis microcephala*), 15,520 (*H. incana*), 16,670 (*H. vernonioides*), 47,826 (*H. sericophylla*), and 6498 SNPs (*H. impressinervia*). The mean alignment length per locus of these datasets ranged from 550.32 bp (SE = 0.19) to 568.41 bp (0.28).

### Genetic diversity and differentiation

**Figure 2:**
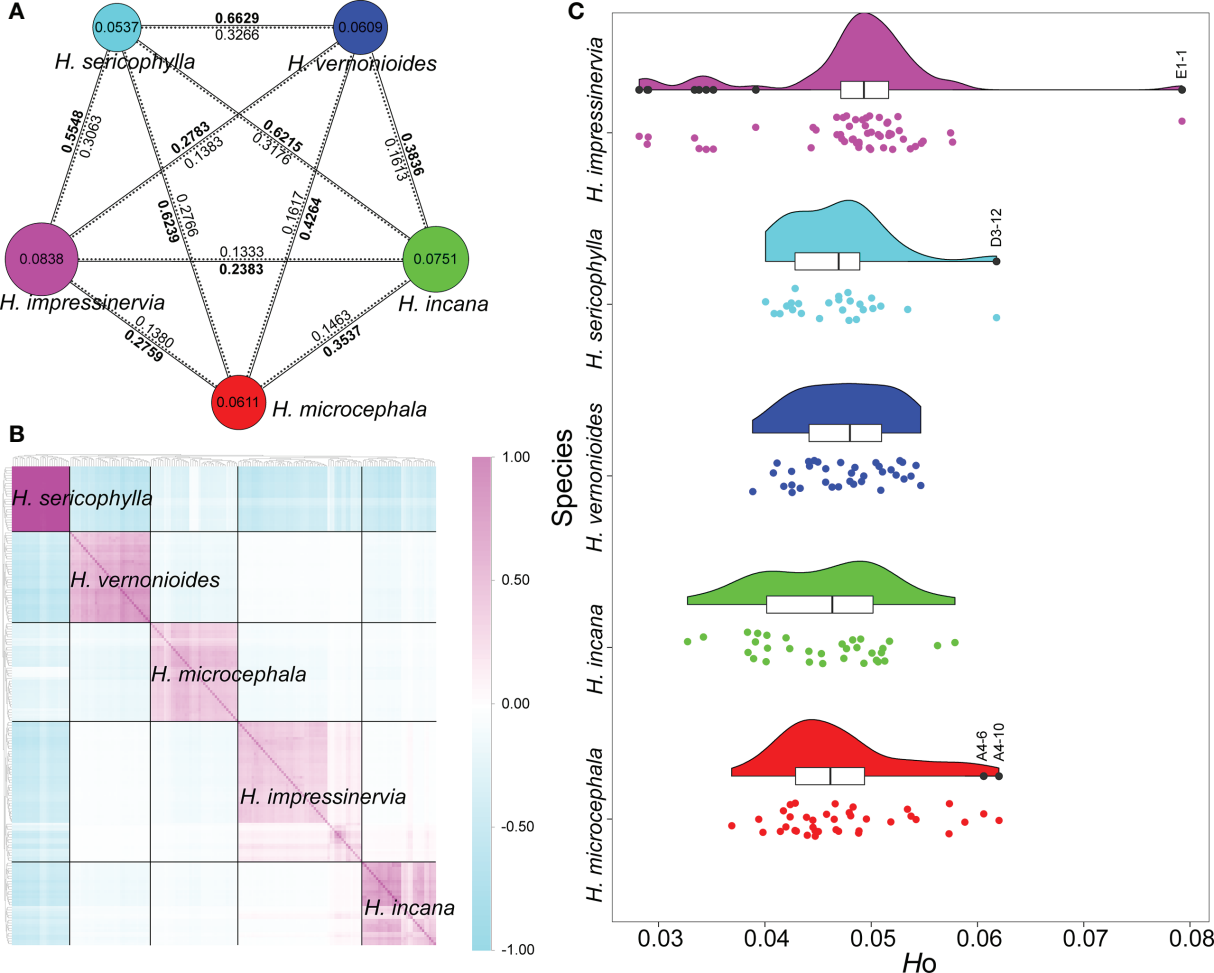
Genetic diversity and genetic differentiation, kinship coefficients, and heterozygosity of *Heteroplexis* species. **(A)** The size of a circle and the value inside denote genetic diversity (π) level. Values of genetic differentiation coefficients (*F*_ST_) are in bold on the solid lines, and values of absolute sequence divergence (*D*_XY_) are on the dotted lines. **(B)** Intra- and interspecific identity-by-state (IBS) kinship coefficients. **(C)** Observed heterozygosity (*H*o) of individuals.

At the species level, the highest genetic diversity (π) was found in *Heteroplexis impressinervia* (0.0838) and the lowest in *H. sericophylla* (0.0537) (**Figure 2A**). The genetic differentiation coefficients (*F*_ST_) ranged from 0.2383 to 0.6629. *H. sericophylla* was the most differentiated from the other species (0.5548–0.6629) and harbored the richest private SNPs (45,987) (**Figure 2A**; **Table S1**). The absolute sequence divergence (*D*_XY_ = 0.1333–0.3266) between species showed a similar pattern to that of *F*_ST_ (**Figure 2A**). At the population level, the populations with the highest π value of its species were A4 (0.0680), B5 (0.0608), C1 (0.0574), D3 (0.0524), and E1 (0.0680), each of which was the northernmost population of its species, except E1 (**Table S1**). Moreover, *H*_O_ and *H*_E_ ranged from 0.0487 to 0.0531 and from 0.0522 to 0.0827, respectively. Of the 25 populations, 18 (72%) had a lower *H*_O_ value than the *H*_E_ value (**Table S1**), and 21 (84%) exhibited a positive *F*_IS_. These results indicate that inbreeding or self-pollination may occur in most populations. The Tassel analysis showed that the intraspecific IBS kinship coefficients were significantly higher than the interspecific coefficients (**Figure 2B**). In addition, the intraspecific kinship coefficients of *H. sericophylla* were higher than those of the other four species. Low interspecific kinship coefficients were observed between *H. impressinervia* and *H. incana*, especially between the individuals of E1 and E6 populations and those of B4 and B5 populations. Several individuals showed a very high heterozygosity, such as A4-10 and A4-6 in *H. microcephala*, D3-12 in *H. sericophylla*, and E1-1 in *H. impressinervia* (**Figure 2C**).

### Phylogenetic inference and genetic structure

**Figure 3:**
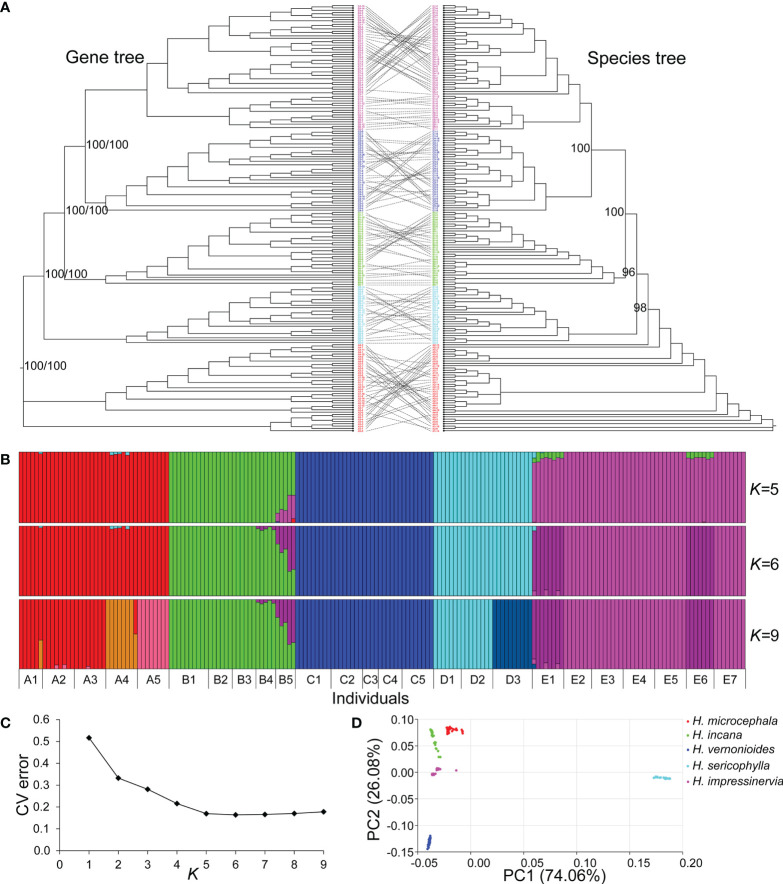
Phylogenetic relationships and genetic structure of species in the genus *Heteroplexis*. **(A)** Gene tree reconstructed based on the maximum likelihood (ML) method and species tree reconstructed based on the SVDQuartets method. **(B)** Population structure analysis using Admixture. **(C)** Cross-validation (CV) errors for *K* = 1–9. **(D)** The first two dimensions of principal component analysis (PCA) based on genome-wide SNP data.

The main clades of the phylogenetic topologies inferred from the gene tree and species tree were consistent, and they all received high bootstrap support (> 95) (**Figure 3A**). These phylogenetic relationships overlapped with present species boundaries. Specifically, H. microcephala was the most rooted clade, while H. sericophylla subsequently diverged, and H. vernonioides and H. impressinervia formed sister clades and shared a common node with H. incana. However, individuals of a same population had closer phylogenetic relationships at the tip of the gene tree.

The Admixture analysis showed that *K* = 6 was the optimal genetic cluster number for the 184 individuals of *Heteroplexis* (**Figure 3B**). Clearly, the five species represented independent genetic lineages. Compared with *K* = 5, two populations of *H. impressinervia* (E1 and E6) and one population of *H. incana* (B5) formed new genetic lineages when *K* = 6. The CV error increased gradually with *K* increasing from 6 to 9, and a new genetic lineage was generally formed by a population. For example, A4, A5, and D3 were distinguished from the other populations of its species when *K* = 9 (**Figure 3C**). However, the B5 population always maintained a substantial admixture. PCA analysis revealed a similar genetic structure, with PC1 and PC2 explaining 74.06% and 26.08% of the total genetic variance, respectively (**Figure 3D**).

### IBD

**Figure 4:**
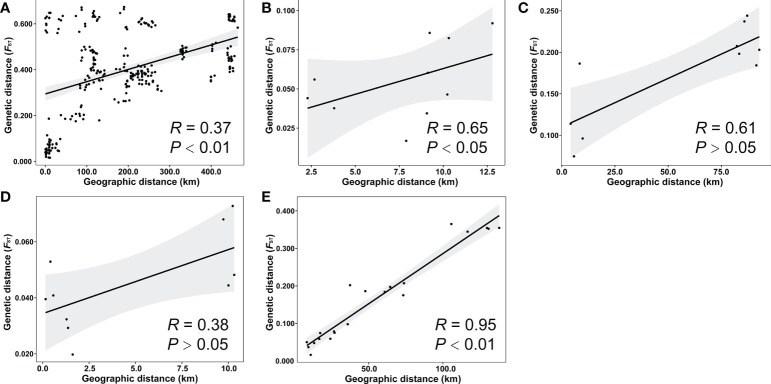
Isolation by distance analysis. Mantel test of the relationship between the genetic and geographic distances among the 25 populations of all species **(A)**, five populations of *H microcephala* **(B)**, five populations of *H incana* **(C)**, five populations of *H vernonioides* **(D)**, and seven populations of *H impressinervia* **(E)**.

The *F*_ST_ values ranged from 0.0161 to 0.6734 and the geographic distances ranged from 0 to 464.2 km among populations of all species (**Figure S1**). The Mantel test showed that there was significant IBD among all 25 populations (*R* = 0.37, *P* < 0.01, **Figure 4A**). For a specific species, the independent Mantel tests revealed significant IBD among the populations of *H. microcephala* (*R* = 0.65, *P* < 0.05, **Figure 4B**) and *H. impressinervia* (*R* = 0.95, *P* < 0.01, **Figure 4E**), but no significant IBD among the populations of *H. incana* (*R* = 0.61, *P* > 0.05, **Figure 4C**) and *H. vernonioides* (*R* = 0.38, *P* > 0.05, **Figure 4D**).

### Interspecific gene flow

**Figure 5:**
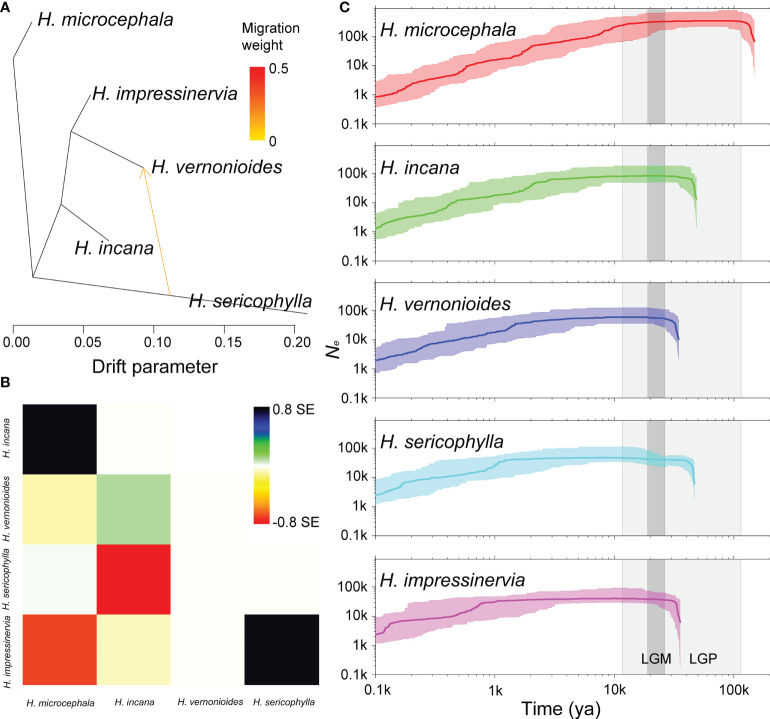
Interspecific gene flow and demographic history of *Heteroplexis.* One gene flow event inferred by Treemix **(A)** and the corresponding residual plot **(B)**. **(C)** Estimates of the effective population size (*N_e_*) using Stairway Plot. Thick and thin light lines represent the median and the 95% pseudo-confidence intervals (CIs) defined by the 2.5% and 97.5% estimations. The light and dark gray shaded areas indicate the last glacial period (LGP) and the last glacial maximum (LGM), respectively. The ya means years ago.

The OptM determined the optimal migration model as m = 1, suggesting that one migration event might has occurred in the five species of *Heterosiphilic* (**Figures 5A, B** and **Figure S1**). With the model of m = 1, we detected gene flow from *H. sericophylla* to *H. vernonioides*, which was confirmed by the significant *D* value in *D*-statistic (**Table 1**). Gene flow between *H. sericophylla* and *H. vernonioides* may has occurred as well when *H. impressinervia* was P1, although the *D* value (0.044) was not significant (*P* = 0.134). However, the two significant (*P* < 0.001) gene introgression events between *H. impressinervia* and *H. incana* (0.172) and between *H. impressinervia* and *H. sericophylla* (0.091) shown by *D* value were undetected in Treemix. Overall, these results suggest that there were a few significant historical gene flows among the *Heterosiphilic* species.

**Table 1:** ABBA-BABA statistic for the five species of *Heteroplexis*.

P1	P2	P3	*D*	Z-score	*P*	f4-ratio
*H. vernonioides*	*H. impressinervia*	*H. incana*	0.172	8.063	< 0.001	0.079
***H. incana***	***H. vernonioides***	***H. sericophylla***	**0.118**	**3.911**	**< 0.001**	**0.010**
*H. incana*	*H. impressinervia*	*H. sericophylla*	0.091	4.260	< 0.001	0.007
*H. impressinervia*	*H. vernonioides*	*H. sericophylla*	0.044	1.499	0.134	0.003

*H. microcephala* is outgroup. The Gene flow event in bold was consistent with Treemix detection.

### Demographic history

We found that the *N_e_* of all five species of *Heterosiphilic* had undergone expansion, stabilization, and continuous decline in history (**Figure 5C**). First, the *N_e_* of *H. microcephala* expanded before the last glacial period (LGP, about 148 kya). Subsequently, the *N_e_* of *H. incana* and *H. sericophylla* expanded in 46–48 kya, followed by *H. vernonioides* and *H. impressinervia* in 34–35 kya during the LGP. After the Holocene (about 12 kya), the *N_e_* of all species except *H. microcephala* experienced a period of stability until about 4 kya. After that, the *N_e_* of all *Heterosiphilic* species entered a collapse period.

## Discussion

### Clear phylogeographic structure among species of the genus *Heteroplexis*

As an endemic genus of China, the genus Heteroplexis has been recorded in many authoritative plant databases or books. However, due to the difficulties in phenotypic identification, the classification of Heteroplexis species is still not widely recognized, although it has been more than 20 years since the last species update (Liang, 1994). For instance, only H. microcephala, *H. sericophylla* and *H. vernonioides* were included in the Flora of China (Chen et al., 2011b). The Plant List included five Heteroplexis species, but only the names of *H. impressinervia* and *H. incana* in “Accepted” status, while the other three were in “Unresolved” status (https:///www.theplantlist.org/). Five Heteroplexis species were included in Tropicos database, but *H. impressinervia* and *H. incana* were not considered to be “Legitimate” (https://www.tropicos.org/). In addition, a previous study by Hu (2015) suggested combining *H. impressinervia, H. incana* and *H. sericophyll* into a single species, as some key morphological traits (e.g., leaf vein) that had been used to distinguish species may be phenotypic variation between populations of the same species. Therefore, the phylogeographic study of the genus Heteroplexis may provide valuable information to clarify the relationships among species.In this study, we used the large-scale genomic loci generated by RADseq to infer the phylogenetic relationships for the first time in the genus Heteroplexis. The reconstructed gene tree and species tree showed that the sympatric / parapatric species *H. microcephala* and *H. sericophylla*, and the allopatric species *H. incana, H. vernonioides* and *H. impressinervia* have stable phylogenetic relationships as independent species. As in some previous studies (Wang et al., 2017), although our phylogenetic trees also lack outgroups, the results can still be used as a reference for species relationships. In addition to phylogenetic analysis, genetic structure analysis confirmed a strong phylogeographic structure among the five species, and the optimal K value (6) implies that there may be a sub-structure within the H. impressinervia species. Finally, the FST and DXY values indicate high levels of genetic differentiation between species. These genomic signals demonstrate a clear phylogeographic structure among the Heteroplexis species, which is consistent with current taxonomic boundaries.

The IBD model assumes that when geographic distance increases, gene flow between populations decreases, and genetic differentiation between populations is positively correlated with geographic distance under the effect of genetic drift (Bohonak, 2002). The clear phylogeographic structure of karst plants is attributed to IBD, which has been highlighted in previous studies (Gao et al., 2015; Tseng et al., 2019). Similarly, the significant global and regional IBD signals indicate strong geographic isolation between the *Heteroplexis* species. Considering that the pollination of *Heteroplexis* species relies mainly on insects, such as *Eristalis ceralis* Fabricius and *Vespa ducalis* Smith, with very limited pollination distances, and that most species are distantly distributed, gene flow between populations or species should be limited. In addition, such flowering biological characteristics of *Heteroplexis* as short life span of a single flower (3 days) or an inflorescence (5 days), short pollen viability period (18 h), short stigma receptivity period (48 h), and flowering asynchronism between a few species (Shi et al., 2015) further limit pollen spread and exacerbate geographic isolation. Thus, long-term restricted gene flow as a result of strong geographic isolation may have exacerbated genetic drift and differentiation and eventually led to the formation of the clear phylogeographic structure among the *Heteroplexis* species.

In contrast to the restricted current gene flow, both Treemix and ABBA-BABA analyses indicated significant historical gene flow events among the *Heteroplexis* species (**Figure 5**). One possible explanation is that these gene flow events occurred at the beginning of species divergence, when the *Heteroplexis* ancestors had a wider range than today for gene flow. However, geological activities and climate change led to the present fragmented populations (Zhang et al., 2021b; Ke et al., 2022). In addition, if current allopatric species have experienced migration events, gene flow events may have taken place when they were sympatric (Sun et al., 2015). Notably, the gene introgression between *H. incana* and *H. impressinervia*, which was detected by *D*-statistic, retained substantial admixture in the intraspecific populations, such as B5. The recent gene introgression events may be caused by species differentiation, rapid radiation, and high hybridization (Ye et al., 2019; Liveri et al., 2020). Overall, *H. incana* shares approximately equal genetic similarity with both sister species *H. impressinervia* and *H. vermonioides.*

### Demographic history of *Heteroplexis* species

Phylogeographic information of species can be used to describe potential phylogeoregions (Lin et al., 2021; Ye et al., 2021). Taking into consideration its current geographic distribution pattern, we hypothesized that the genus *Heteroplexis* was originally distributed in northeastern Guangxi (*H. microcephala* and *H. sericophylla*) and then migrated continuously southward or southwestward to central Guangxi (*H. incana* and *H. impressinervia*) and southwestern Guangxi (*H. vernonioides*). To support our hypothesis, we inferred the demographic histories of the five species. The order of the first expansion times of *Ne* for *H. microcephala* (148 kya), *H. incana* and *H. sericophylla* (46–48 kya), and *H. vernonioides* and *H. impressinervia* (34–35 kya) (**Figure 5C**) supports our inference of the phylogenetic relationships among *Heteroplexis* species, although these estimated times of species divergence remain to be calibrated. Furthermore, for a specific species, the population with the highest genetic diversity was found to be the most northerly distributed. One exception was E1, which was the second most northerly distributed among all populations of its species. This pattern of genetic diversity distribution may be attributed to the founder effect, which can result in lower genetic diversity in populations that colonize later (Díaz-Pérez et al., 2008). These results suggest north-to-south migration of both the species and populations of *Heteroplexis*. It is known that many plants on the Asian continent underwent north-to-south migration during the LGP (Chen et al., 2011a). The main driving forces of the migrations are likely to be the LGP induced climatic changes such as falling temperature and precipitation and weakening monsoons (Zhisheng et al., 2001). In this case, *Ne* was maintained at a steady state during the LGP for all *Heteroplexis* species, indicating that migration was effective in stabilizing populations. The response of *Heteroplexis* to the LGP is similar to those of such genera as *Cycas* (Tao et al., 2021), *Primulina* (Ke et al., 2022), and *Rhododendron* (Zhang et al., 2021b), suggesting that the karst mountains in Guangxi may be a glacial refugium for *Heteroplexis*. It is suggested to reconstruct the biogeographic history of *Heteroplexis* at a finer genomic scale and incorporate other evidence, such as fossil information (Yan et al., 2021), to test the above mentioned hypothesis.

### Genetic diversity and conservation suggestions

Genetic diversity provides important information for the design of conservation programs (Rossetto et al., 2021). With large-scale molecular markers generated by RADseq, previous studies showed considerable variations in genetic diversity level in endangered plants, such as *Viola uliginosa* Besser (π = 0.04395) (Lee et al., 2020), Rhododendron cyanocarpum (Franch.) Franch. ex W.W. Sm. (0.0702) (Liu et al., 2020), Prunus mongolica Maxim. (0.339) (Zhang et al., 2021a), and several species of genus Cycas (0.000030.00262) (Tao et al., 2021). The nucleotide polymorphisms in Heteroplexis species were 0.05370.0838, higher than that (0.0347) of the recently reported Myripnois dioica Bunge in Asteraceae (Lin et al., 2021) Our results suggest that the genetic diversity of *Heteroplexis* species was not as low as expected, although their habitat was fragmented and their population size was small. On the one hand, the significant historical gene flows led to the high genetic diversity (Zhang et al., 2021b). On the other hand, the *Ne* of each *Heteroplexis* species was maintained at a stable level for a long time in history according to the Stairway Plot (**Figure 5C**). Thus, the genetic variation of the population itself can accumulate continuously and effectively.

However, for species with long-term distribution in narrow areas and small population sizes, they would face a high risk of genetic diversity loss (Zhang and Jiang, 1999; Bortoluzzi et al., 2020). *Heteroplexis* plants are outcrossing and partially selfing (Shi et al., 2015), and the *F*_IS_ values indicate inbreeding in most of the populations (**Table S1**). As such, attention needs to be paid first and foremost to inbreeding depression. Then, it is important to note that there has been a recent *Ne* collapse in each species. The population size of *Heteroplexis* is lower in densely vegetated habitats than in sparsely vegetated habitats (e.g., bare rocks). According to previous studies, the climate has stabilized after entering the Holocene, and the populations of many species have been gradually recovering or exploding (Miao et al., 2021; Ke et al., 2022). We speculate that the population recovery of other species has led to a smaller ecological niche of *Heteroplexis* species and thereby a smaller size of their population. Besides, the negative impact of habitat fragmentation on population size due to the recent increase in human activities needs to be given attention to (Chi and Hung, 2010; Yuan and Wei, 2015; Antonelli et al., 2018; Zhang et al., 2020), especially the continuous decline of *N_e_* in *Heteroplexis* species since 4 kya. A recent study on a karst plant species, *Begonia luzhaiensis*, in Guangxi reported a coincidence between the periods of increasing human activity and declining population of the species (Tseng et al., 2019). This is strikingly similar to the case of *Heteroplexis*, implying that the karst plant species in Guangxi have been generally experiencing population decline. Similar declines were found in several common forest birds in southern China, revealing that human disturbance is the main cause behind the current unprecedented rate of biodiversity loss (Dong et al., 2022). It is crucial to note that the decline of *N_e_* in *Heteroplexis* seems to continue. As far as we know, the Bilianfeng population, where the model specimen of *H. microcephala* was discovered, has disappeared (Shi et al., 2016). If the Ne of a species is too small, its adaptive potential would be very low, and it would face a very high extinction risk (Jin et al., 2021). Considering these potential threats, we strongly suggest to strengthen the protection of the endangered endemic karst plants in Guangxi. Besides in situ conservation of the karst landscapes, ex situ Heteroplexis conservation should be given the priority. Individuals from the founder population or individuals with high heterozygosity in each population should be used as ex situ conservation objects to maintain high genetic diversity of the population.

## Conclusions

In summary, we revealed a clear phylogeographic structure among *Heteroplexis* species which was consistent with the current species boundaries, indicating that the five *Heteroplexis* species were all independent species. Long-term restricted gene flow as a result of strong geographic isolation by the karst mountains exacerbated genetic drift and differentiation, possibly leading to the clear phylogeographic structure. However, the complex geography of the karst mountains provided refuges for *Heteroplexis* during the LGP. We inferred that *Heteroplexis* species first originated in northeastern Guangxi before the LGP, then migrated continuously to the southwestern Guangxi, and formed the current species distribution. The long-term stable *Ne* and significant historical interspecific gene flow contributed to the high level of genetic diversity. However, the recent decline of *Ne*, widespread inbreeding within populations, and restricted gene flow suggest that *Heteroplexis* is still facing a high risk of genetic diversity loss. Therefore, there is still a need to strengthen the conservation of *Heteroplexis*.

## Data availability statement

The original contributions presented in the study are included in the article/**Supplementary Material**. The raw data of RADseq were uploaded to NCBI with the SRA accession number of PRJNA850667. Further inquiries can be directed to the corresponding authors.

## Author contributions

YS, LD, and XW conceived the study and designed the experiments. XZ, HL, and HJ conducted data analysis. HL, LD, and HJ performed the sample collection. XZ wrote the manuscript. MK guided the data analysis and revised the manuscript. All authors contributed to the article and approved the submitted version.

## Funding

This work was supported by the fund of National Natural Science Foundation of China (31960276) and Chinese Academy of Sciences “Light of West China” Program (2018).

## Acknowledgments

We thank Dr. Huiqin Yi of South China Botanical Garden, Chinese Academy of Sciences, for her help in bioinformatics analysis.

## Conflict of interest

The authors declare that the research was conducted in the absence of any commercial or financial relationships that could be construed as a potential conflict of interest.

## Publisher’s note

All claims expressed in this article are solely those of the authors and do not necessarily represent those of their affiliated organizations, or those of the publisher, the editors and the reviewers. Any product that may be evaluated in this article, or claim that may be made by its manufacturer, is not guaranteed or endorsed by the publisher.
