# Supplementary material for: Phylogeographic structure of Heteroplexis (Asteraceae), an endangered endemic genus in the limestone karst regions of southern China
Source: Front Plant Sci. 2022 Oct 27;13:999964. doi: 10.3389/fpls.2022.999964 (PMC9647136; doi:10.3389/fpls.2022.999964)
Supplement: Supplementary file 1 [file DataSheet_1.zip › Supplementary_Material/Supplementary_Material.docx]

Supplementary Material

# Supplementary Tables

**Table S1** Main genetic parameters at species and population level.

**Table S2** Sequencing statistics of 184 samples.

**Table S3** Sequence characteristics of three data sets.

# Supplementary Figures


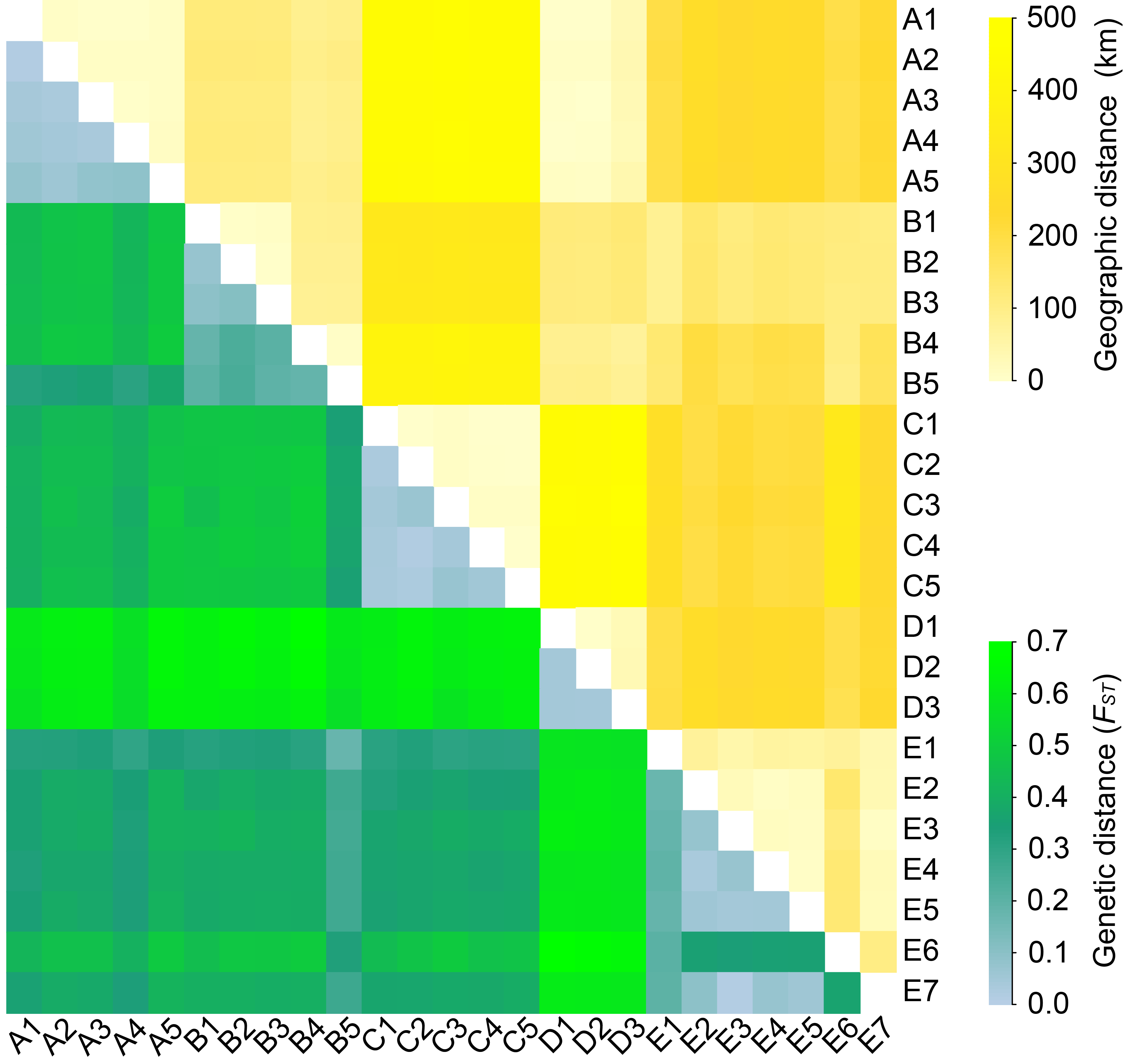


**Fig. S1.** Genetic and geographic distances between populations.


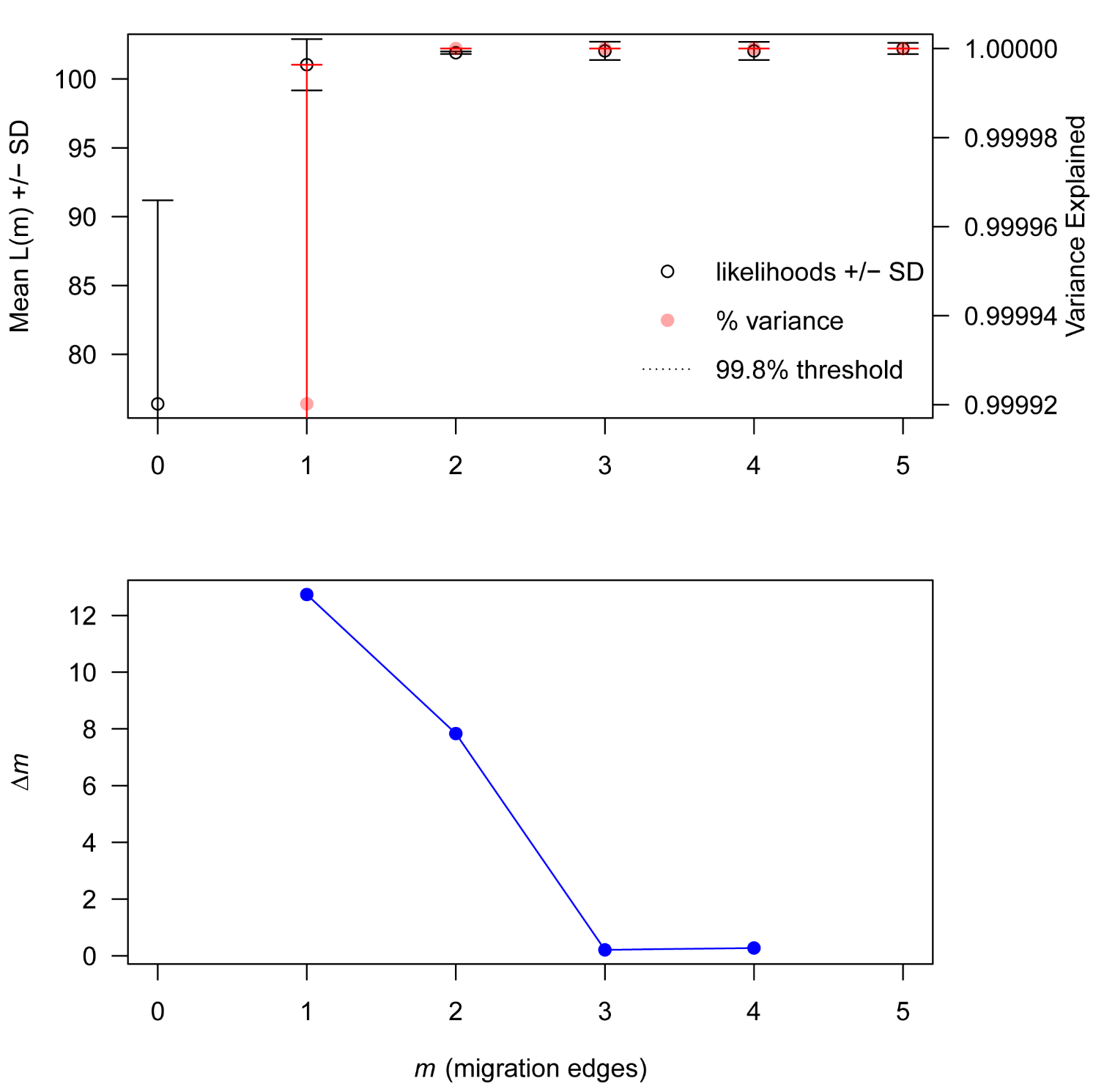


**Fig. S2.** Detection of the optimal migration model using OptM.
